# Postoperative Effects of Celecoxib on Opioid Use and Pain Control After Benign Oropharyngeal Surgery

**DOI:** 10.1002/lary.70530

**Published:** 2026-03-29

**Authors:** Alana Platukus, Praneet Kaki, Anastasia Kaffenes, Udeerna Tippabhatla, Jared Robinson, Jaehee Kim, Erin Creighton, Meghan Crippen, Maurits Boon, Colin Huntley

**Affiliations:** ^1^ Sidney Kimmel Medical College at Thomas Jefferson University Philadelphia Pennsylvania USA; ^2^ Department of Otolaryngology & Sleep Medicine Thomas Jefferson University Hospital Philadelphia Pennsylvania USA

**Keywords:** adenoidectomy, celecoxib, opioid usage, pain score, tonsillectomy, uvulopalatopharyngoplasty

## Abstract

**Objectives:**

To establish how celecoxib use after tonsillectomy and adenoidectomy (T&A) and modified uvulopalatopharyngoplasty (UPPP) affect pain scores and opioid consumption.

**Methods:**

Patients undergoing T&A or UPPP with tonsillectomy (May–October 2024) completed surveys on postoperative days (POD) 0, 1, 5, and 10 to assess pain scores and opioid use. All patients received a multimodal pain regimen, and cohorts were based on the prescription of celecoxib. The noncelecoxib cohort was a historical control group obtained via study data from Butkus et al. (December 2020–January 2023)*.* Wilcoxon rank‐sum and chi‐squared tests were performed using R Studio.

**Results:**

Eighty patients were included (mean 33.0 years [SD = 12]), 66% female; 34% male; 55% Caucasian. The celecoxib cohort (*N* = 38) consumed an average of 66.2 mL (SD = 62.1) of opioids, and the non‐celecoxib cohort (*N* = 42) received an average of 118.4 mL (SD = 91.6) (*p* = 0.021). There were no significant differences in pain scores reported on POD 1, 5, or 10 (*p* = 0.5, *p* = 0.2, *p* = 0.6, respectively).

**Conclusion:**

The addition of postoperative celecoxib resulted in a significant decrease in mean opioid consumption. Our data also demonstrate that postoperative pain was controlled similarly based on patient reported pain scores, regardless of celecoxib consumption. Further investigation is warranted with larger sample sizes to determine the efficacy of postoperative celecoxib use.

**Level of Evidence:**

3.

## Graphical abstract

This study evaluated how the addition of celecoxib to a multimodal pain regimen following tonsillectomy + adenoidectomy, or uvulopalatopharyngoplasty (UPPP) + tonsillectomy affects postoperative opioid use and pain control. Patients who received celecoxib consumed significantly fewer opioids compared to the historic control group, with similar postoperative pain scores across both cohorts on postoperative days 1–10. These findings suggest that celecoxib may help to reduce opioid requirements without compromising analgesia after benign oropharyngeal surgeries.

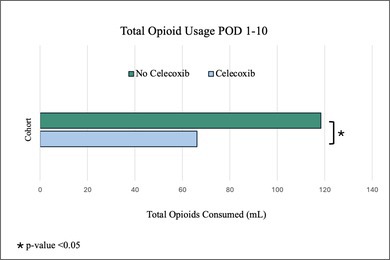

## 1 Introduction

The opioid epidemic remains a pressing public health crisis in the United States. Last year, approximately 82,138 drug overdose deaths were recorded by the CDC, of which 52,335 were opioid related [1]. Beyond the devastating effects on people, the opioid crisis carries significant economic consequences, with the U.S. Congress Joint Economic Committee estimating a $1.5 trillion burden in 2020 attributable to the opioid epidemic as a whole, an increase of 37% since 2017 [2, 3]. Among the many contributors to opioid exposure, perioperative prescriptions for pain management play a significant role, with persistent postoperative opioid use continuing to be one of the most common and adverse long‐term risks of surgery [4]. The COVID‐19 pandemic further exacerbated this trend by delaying elective procedures, leading to increased opioid prescriptions and usage in the interim [5, 6].

In response to the opioid crisis, healthcare systems have adopted strategies to reduce opioid reliance after surgery, including enhanced recovery after surgery (ERAS) pathways, improved provider education, and increased training on safe administration of analgesics [7]. Multimodal pain regimens, which combine a variety of analgesic medications with different mechanisms of action, have become a widely adopted standard of care [7]. This approach provides effective pain relief while minimizing side effects associated with any single agent [8, 9].

Among nonopioid agents, nonsteroidal anti‐inflammatory drugs (NSAIDs) are widely used in multimodal pain regimens. Celecoxib, a selective COX‐2 inhibitor, has gained increasing attention for its role in postoperative pain control. Studies have demonstrated that celecoxib can reduce opioid consumption and improve recovery following different surgeries [10, 11]. A recent meta‐analysis involving patients undergoing tonsillectomy and adenoidectomy (T&A) showed significantly less postoperative pain in patients who used celecoxib compared with placebo groups [12]. Furthermore, in regard to the side effect profile of celecoxib, one study evaluating children who underwent tonsillectomy showed that celecoxib has not been associated with an increased risk of bleeding compared to acetaminophen postoperatively [13]. While these findings are promising, the effectiveness of celecoxib and influence on opioid consumption after oropharyngeal procedures in adults, particularly after uvulopalatopharyngoplasty (UPPP) with tonsillectomy, remains underexplored.

Given this gap in evidence, our study seeks to evaluate the addition of celecoxib to standard postoperative pain regimens in patients undergoing benign oropharyngeal surgeries, such as T&A and modified UPPP with tonsillectomy. We hypothesize that celecoxib will allow additive benefit to our standard postoperative pain regimen, allowing for decreased patient‐reported pain scores and reduced reliance on postoperative opioids.

## 2 Methods

This prospective cohort study was approved by the institutional review board at Thomas Jefferson University Hospital. Adult patients, ages 18 years and older, who underwent tonsillectomy, T&A, or Tonsillectomy + UPPP between May and October 2024 were enrolled in this study. All procedures were performed by 1 of 3 senior authors (CH, MB, and MC) in the Thomas Jefferson Department of Otolaryngology; all tonsillectomies were extracapsular tonsillectomies, and electrocautery was used in all surgeries. All patients received a postoperative multimodal pain regimen of celecoxib (200 mg twice daily, 28 tablets total), oxycodone (5 mg every 6 h, 150 cc total), gabapentin (300 mg twice daily, 28 capsules total), acetaminophen (640 mg every 6 h, 180 cc total), magic mouthwash (diphenhydramine 12.5 mg/5 mL liquid, aluminum–magnesium hydroxide‐simethicone 200–200‐20 mg/5 mL, lidocaine 2% solution) (5 mL every 4 h), and prednisone taper (40 mg for 3 days, 30 mg for 3 days, 20 mg for 3 days, and 10 mg for 3 days). These medications were all started on the day of surgery, and the prescribed opioids were instructed to be consumed on an “as needed” basis. If opioids were used in total, refills were provided in the celecoxib cohort upon patient request for uncontrolled pain, in which five additional 5 mg oxycodone tablets (rather than liquid oxycodone) were prescribed. Patients were not prescribed proton pump inhibitors (PPIs) or any additional gastroprotective therapy. The control cohort for this study was obtained retrospectively using the study data from Butkus et al. [14] The historic control cohort underwent similar procedures between December 2020 and January 2023 and received the same postoperative pain regimen consisting of opioids, gabapentin, acetaminophen, magic mouthwash, and prednisone taper, without the prescription of celecoxib. The majority of opioids prescribed in the control cohort consisted of oxycodone (in liquid or tablet form), and two patients were prescribed hydrocodone.

Chart review was conducted on all patients, and collected data included demographic information, medical comorbidities, smoking history, and drug history including marijuana, cocaine, and ketamine. Postoperative courses, including readmission or return to the operating room (OR), were also collected. Patients were excluded if they had contraindications to celecoxib, including bleeding disorders or a history of postoperative hemorrhage. Those with comorbid opioid use disorder, current opioid use, chronic pain syndromes, or pregnancy were also excluded. Additionally, patients who did not take celecoxib as prescribed throughout the postoperative 10 day study period were excluded. Patients were excluded from the historical control set if they underwent any other procedure other than T&A or UPPP with tonsillectomy.

**FIGURE 1:**
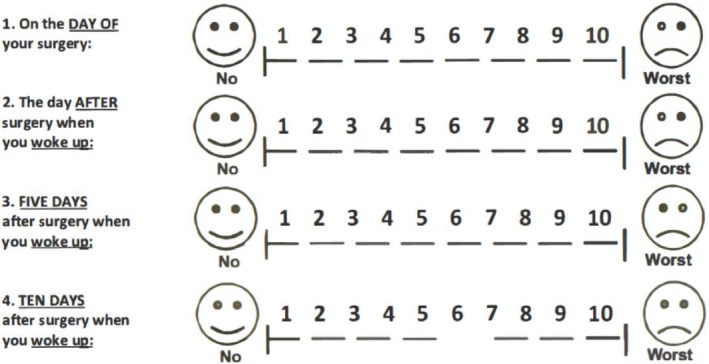
Pain score visual analog scale. Patients used the visual analog scale (1–10) to report pain scores on postoperative days 0, 1, 5, and 10. [Color figure can be viewed in the online issue, which is available at www.laryngoscope.com]

**FIGURE 2:**
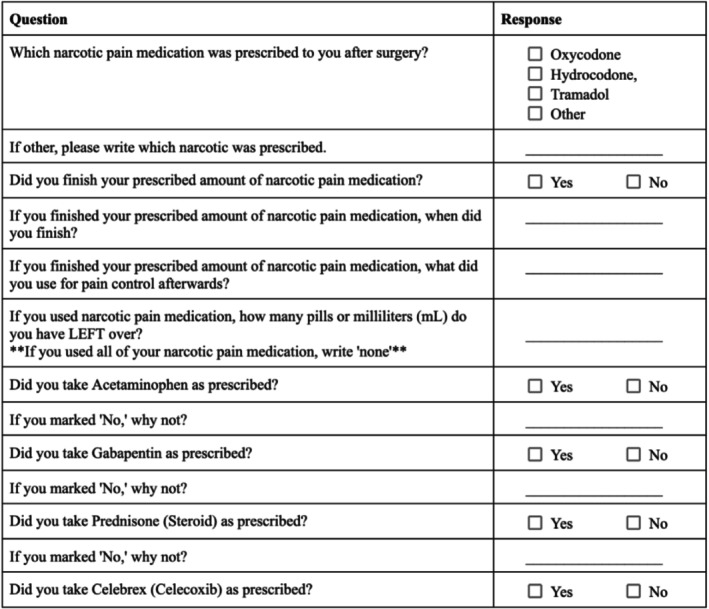
Postoperative survey questions regarding opioid use. Patients answered the questions to report their opioid usage on postoperative day 10.

Patients were sent electronic RedCap surveys via email on postoperative days 0, 1, 5, and 10 to report pain on each respective day. Patients reported their pain score on a visual analog scale from 1 to 10, with a score of 1 indicating no pain to very minimal pain, and 10 indicating severe pain (Figure 1). On postoperative day 10, in addition to their pain score, patients also reported total opioid usage, opioid amount remaining (asked to measure in a graduated medication cup), and whether all medications were taken according to prescription instructions (Figure 2). Patients who consumed their entire opioid prescription also indicated if they requested and received a refill. All opioid amounts were converted to morphine milligram equivalents (MME) for standardization and further analysis. One dose of prescribed opioids was defined as 5 mg or 7.5 MME, which was consumed as 5 mL of liquid oxycodone.

General descriptive statistics were used to report on the demographics and baseline characteristics of the cohort. Continuous variables were reported as mean (standard deviation), and categorical variables were reported as frequency (percentage). Wilcoxon rank‐sum tests were used to compare continuous variables between patients who did and did not receive celecoxib; Fisher's exact and chi‐squared tests were performed to analyze categorical data. An alpha level of 0.05 was used for all tests. Statistical analyses were performed using R Studio Version 2023.03.0.

## 3 Results

A total of 80 patients (mean 33.0 years [SD = 12], 66% female; 34% male; 55% Caucasian; 45% non‐white including African American, Hispanic, Asian, and Pacific Islander) were included in this study. There were 38 (47.5%) patients in the treatment cohort who received celecoxib, and 42 (52.5%) in the control cohort that did not. Demographic and baseline characteristics are described in Table 1. In the celecoxib cohort, three patients had postoperative bleeding episodes, two of which required intervention in the OR. None of the patients, including the aforementioned patients who experienced a postoperative bleeding event, were readmitted to the hospital postoperatively.

**TABLE 1:** Descriptive characteristics of treatment cohorts.

Characteristic	*N*	Overall, *N* = 80^a^	Celecoxib, *N* = 38^a^	No celecoxib, *N* = 42^a^	*p* ^b^
Age	80	33.6 (12.5)	33.9 (13.1)	33.4 (12.1)	> 0.9
Sex	80				0.6
Female		53 (65%)	24 (63%)	29 (67%)	
Male		27 (34%)	14 (37%)	13 (31%)	
Race	80				> 0.9
Non‐White		36 (45%)	17 (45%)	19 (45%)	
White		44 (55%)	21 (55%)	23 (55%)	
Procedure	80				0.8
T ± A		66 (82%)	31 (82%)	35 (83%)	
Tonsillectomy and UPPP		14 (18%)	7 (18%)	7 (17%)	

Abbreviations: POD, postoperative day; T ± A, tonsillectomy and adenoidectomy; UPPP, uvulopalatopharyngoplasty.

^a^ Mean (SD); *n* (%).

^b^ Wilcoxon rank sum test; Pearson's chi‐squared test; Fisher's exact test.

### 3.1 Pain Regimen Adherence

A total of four patients in the celecoxib cohort were excluded because they did not take any of the prescribed celecoxib, either due to gastrointestinal intolerance (one patient) or because celecoxib was not prescribed despite no prior documented contraindications (three patients). Consumption of all other prescribed medications (gabapentin, acetaminophen, magic mouthwash, and prednisone) was generally well followed. In the celecoxib cohort, two patients (5.3%) reported not taking gabapentin as prescribed because they felt they did not need it based on pain level. In the noncelecoxib cohort, six patients (14.2%) reported not taking acetaminophen as prescribed. Of these patients, one was due to an acetaminophen allergy, and five patients reported not needing it based on their pain level. In the noncelecoxib cohort, four patients (9.5%) did not take gabapentin as prescribed. One patient indicated this was due to a “burning sensation” side effect, and the other three reported it was not needed based on pain score.

### 3.2 Opioids Consumed

The noncelecoxib cohort was prescribed an average of 177.1 mL (SD = 71.7) of opioids, compared to 147.4 mL (SD = 13.1) across the celecoxib cohort (*p* = 0.3). The noncelecoxib cohort consumed, on average, more opioids than the celecoxib cohort (118.4 vs. 66.2 mL, *p* = 0.021), consuming 66.9% of prescribed opioids compared to 44.9% of opioids prescribed in the celecoxib group. The noncelecoxib cohort had 58.1 mL (SD = 54.7) of opioids left over, and the celecoxib cohort had 70.4 mL (SD = 62.6) left over. There were no significant differences in the amount of requested or prescribed medication refills, and 82% of all patients required no refills (Table 2).

**TABLE 2:** Opioid consumption by cohort.

	*N*	Overall, *N* = 80^a^	Celecoxib, *N* = 38^a^	No celecoxib, *N* = 42^a^	*p* ^b^
Total opioids used (mL)	62	99.9 (85.6)	66.2 (62.1)	118.4 (91.6)	0.021
Opioids prescribed (mL)	73	163.7 (55.5)	147.4 (13.1)	177.1 (71.7)	0.3
Opioids left (mL)	63	62.4 (57.4)	70.4 (62.6)	58.1 (54.7)	0.4
MME prescribed	77	253.0 (100.4)	219.6 (29.6)	280.9 (127.3)	0.090
MME used	66	165.3 (139.1)	113.5 (88.4)	194.9 (154.3)	0.025
MME left over	66	90.9 (85.0)	99.6 (92.0)	86.0 (81.4)	0.5
Number of refills	80				> 0.9
0		66 (82%)	32 (84%)	34 (81%)	
1		11 (14%)	5 (13%)	6 (14%)	
2		2 (2.5%)	1 (2.6%)	1 (2.4%)	
3		1 (1.3%)	0 (0%)	1 (2.4%)	

Abbreviation: MME, morphine milligram equivalents.

^a^ Mean (SD); *n* (%).

^b^ Wilcoxon rank sum test; Pearson's chi‐squared test; Fisher's exact test.

### 3.3 Patient‐Reported Pain Scores

Patient‐reported pain scores were significantly reduced on postoperative day 0 in the celecoxib cohort compared to the noncelecoxib cohort (5.2 vs. 6.6, *p* = 0.035). There were no significant differences in pain scores between the two cohorts on postoperative days 1 (6.1 vs. 6.4, *p* = 0.5), 5 (6.7 vs. 6.0, *p* = 0.2), or 10 (3.8 vs. 3.5, *p* = 0.6), respectively (Table 3).

**TABLE 3:** Self‐reported postoperative pain score by treatment cohort.

POD	*N*	Overall, *N* = 80^a^	Celecoxib, *N* = 38^a^	No celecoxib, *N* = 42^a^	*p* ^b^
0	80	6.0 (2.6)	5.2 (2.8)	6.6 (2.4)	0.035
1	80	6.2 (2.4)	6.1 (2.3)	6.4 (2.6)	0.5
5	70	6.3 (2.4)	6.7 (2.3)	6.0 (2.4)	0.2
10	64	3.6 (1.9)	3.8 (1.9)	3.5 (1.9)	0.6

Abbreviation: POD, postoperative day.

^a^ Mean (SD); *n* (%).

^b^ Wilcoxon rank sum test; Pearson's chi‐squared test; Fisher's exact test.

### 3.4 Opioids Consumed and Postoperative Pain Stratified by Surgery

Opioid consumption and postoperative pain scores were further analyzed by surgical procedure (Tonsillectomy ± Adenoidectomy [T ± A] and Tonsillectomy + UPPP). There were no statistically significant differences in mean total opioid consumption between groups (T ± A: 101.6 mL, Tonsillectomy + UPPP: 91.2 mL, *p* = > 0.9) (Table 4). Similarly, total MME used did not significantly differ by surgical type (T ± A: 167.2 mg, Tonsillectomy + UPPP: 156.0 mg, *p* = 0.8). There were no significant differences in pain scores by surgical group on any postoperative days: POD 0 (*p* = 0.6) POD 1 (*p* = 0.6), POD 5 (*p* = 0.1), POD 10 (*p* = 0.3) (Table 5). Between surgical groups, the amount of opioids prescribed were statistically significant (*p* = 0.039), but there were no statistically significant differences in the amount of opioids used (*p* = > 0.9), opioids left over (*p* = 0.6), MME prescribed (*p* = 0.3), MME used (*p* = 0.8), or MME left over (*p* = 0.5). The proportion of patients who required no opioid refills was higher in the T ± A group (85%) compared to the Tonsillectomy + UPPP group (71%), although this difference was not statistically significant (*p* = 0.4) (Table 4).

**TABLE 4:** Characteristics and opioid use by procedure type.

Characteristic	*N*	Overall, *N* = 80^a^	T ± A, *N* = 66^a^	Tonsillectomy and UPPP, *N* = 14^a^	*p* ^b^
Celecoxib Y/N	80				0.8
Celecoxib cohort		38 (48%)	31 (47%)	7 (50%)	
No celecoxib cohort		42 (52%)	35 (53%)	7 (50%)	
Age	80	33.6 (12.5)	31.1 (11.3)	45.6 (11.1)	< 0.001
Sex	80				< 0.001
Female		53 (66%)	50 (76%)	3 (21%)	
Male		27 (34%)	16 (24%)	11 (79%)	
Race	80				0.011
Non‐White		36 (45%)	34 (52%)	2 (14%)	
White		44 (55%)	32 (48%)	12 (86%)	
Total opioids used (mL)	62	99.9 (85.6)	101.6 (90.3)	91.2 (58.0)	> 0.9
Opioids prescribed (mL)	73	163.7 (55.5)	167.8 (60.2)	144.6 (13.9)	0.039
Opioids left (mL)	63	62.4 (57.4)	64.2 (57.1)	52.8 (61.3)	0.6
MME prescribed	77	253.0 (100.4)	258.4 (109.1)	228.9 (37.9)	0.3
MME used	66	165.3 (139.1)	167.2 (146.4)	156.0 (99.0)	0.8
MME left over	66	90.9 (85.0)	94.0 (84.8)	75.4 (88.2)	0.5
Number of refills	80				0.4
0		66 (82%)	56 (85%)	10 (71%)	
1		11 (14%)	8 (12%)	3 (21%)	
2		2 (2.5%)	1 (1.5%)	1 (7.1%)	
3		1 (1.3%)	1 (1.5%)	0 (0%)	

Abbreviation: MME, morphine milligram equivalents.

^a^ Mean (SD); *n* (%).

^b^ Wilcoxon rank sum test; Pearson's chi‐squared test; Fisher's exact test.

**TABLE 5:** Self‐reported postoperative pain score by procedure type.

Characteristic	*N*	Overall, *N* = 80^a^	T ± A, *N* = 66^a^	Tonsillectomy and UPPP, *N* = 14^a^	*p* ^b^
Celecoxib Y/N	80				0.8
Celecoxib cohort		38 (48%)	31 (47%)	7 (50%)	
No celecoxib cohort		42 (52%)	35 (53%)	7 (50%)	
Pain score POD 0	80	6.0 (2.6)	6.0 (2.6)	5.6 (3.0)	0.6
Pain score POD 1	80	6.2 (2.4)	6.2 (2.3)	6.3 (3.0)	0.6
Pain score POD 5	70	6.3 (2.4)	6.6 (2.4)	5.2 (2.4)	0.1
Pain score POD 10	64	3.6 (1.9)	3.7 (2.0)	3.0 (1.6)	0.3

Abbreviation: POD, postoperative day.

^a^ Mean (SD); *n* (%).

^b^ Wilcoxon rank sum test; Pearson's chi‐squared test; Fisher's exact test.

## 4 Discussion

This study demonstrates that patients who did not receive postoperative celecoxib in their pain regimen consumed more opioids (194.9 vs. 113.5 MME, *p* < 0.05). Although the celecoxib cohort overall was prescribed a lower amount of opioids compared to the noncelecoxib cohort, patients receiving celecoxib consumed a lower percentage of their prescribed opioids, 44.9% vs. 66.9%, respectively. However, it is important to note that opioid usage was self‐reported by the patients, and while celecoxib may help in reducing opioid intake, the multimodal nature of the prescribed pain regimen used should not be understated. The difference in amount of narcotics prescribed between the cohorts is attributable to evolving prescribing practices following the study results in Butkus et al., in an attempt to minimize leftover household opioids available for diversion or misuse [14]. Of note, the amount of refills did not differ between treatment cohorts in this study. Refills in the celecoxib cohort were not withheld as part of reducing household opioid availability if requested by the patient for uncontrolled postoperative pain.

Given the goal of minimizing access to opioids, it is reasonable to further decrease the amount of opioids prescribed from 150 mL based on our study findings. In the celecoxib cohort, an average of 66.2 mL of oxycodone was used. Although the standard deviation was high (SD = 62.1), demonstrating wide variability between patient pain experiences. The amount of oxycodone routinely prescribed is intended to be decreased in the Otolaryngology department after the results of this study. Although not yet implemented clinically, one proposed reduction strategy is to decrease the total opioid prescription to 125 mL, which helps to minimize remaining opioids, yet cover most patients' needs within the standard deviation. Nonetheless, a study with a larger sample size is needed to more accurately suggest an updated opioid prescribing method.

In this study, the addition of celecoxib did not significantly affect pain scores on postoperative days 1, 5, or 10, or opioid refill rates. The culmination of these results suggests that the addition of celecoxib postoperatively can help to decrease reliance on opioids after benign oropharyngeal surgery while adequately managing pain by either subjectively reducing pain or providing similar relief to opioids alone. When stratified by procedure type, the amount of postoperative opioid use did not differ. Self‐reported pain scores were significantly different on the same day of surgery only in the two treatment cohorts, with no significant difference based on surgery type. It is likely that the significant decrease in pain scores between cohorts on postoperative day 0 is influenced by management in the OR and postanesthesia care unit (PACU), thus potentially confounding the effects of celecoxib on the same day of surgery. Nonetheless, continued investigation is warranted to delineate these differences in PACU management and pain scores, and further emphasizes the need for evaluation of postoperative pain management modalities in oropharyngeal procedures. Ultimately, our results suggest the efficacy of celecoxib use postoperatively. However, the subjective nature of pain measured in our study also demonstrates that celecoxib is unlikely to be the only solution for adequate pain control, and continued investigation is warranted. This study lends insight into important clinical practices to work toward decreasing the public health burden of prescription opioid overuse and the potential for associated adverse effects.

Recent trends show that there has been an overall decrease in opioid prescribing for pain management, with the AMA reporting a greater than 55% decrease of total MME and a greater than 44% decrease in total opioid prescriptions between 2011 and 2020 [15]. Despite this progress, prescription opioids still remain a public health concern due to increasing rates of opioid‐related overdoses and deaths in the United States. One avenue of developing opioid dependence is through the perpetual misuse of prescription opioids for pain control after surgery, including patterns of long‐term opioid use following procedures and unnecessarily high opioid prescription rates. According to previous studies, 8% of opioid‐naive patients were found to have long term opioid use after receiving postsurgical opioid prescriptions, and the risk of long‐term use was directly related to the duration of opioid consumption [16, 17]. Furthermore, it has been shown that socioeconomic status, gender, age, and mental health may also contribute to the risk of developing chronic opioid use after surgery [18]. Previous research has also indicated that opioids are often prescribed far more than are required and at greater amounts than are used by patients [19, 20, 21]. For example, Howard et al. emphasized the phenomenon that the quantity of prescribed opioids was associated with higher reported opioid consumption even after controlling for postoperative pain [20]. This research, taken together, proves a need for analgesic alternatives postoperatively and continued surgeon opioid stewardship.

To address the concern of potential opioid misuse after surgery, recent research across multiple surgical fields has focused on alternative pain management strategies that reduce either the amount of opioids or the duration of opioids needed. For example, a study comparing opioids versus nonopioid pain management in outpatient breast surgeries found the combination of celecoxib and gabapentin to be superior to opioid pain control in several aspects [22]. Specifically, the nonopioid group required significantly less rescue analgesia and antiemetics in the PACU and had a shorter PACU stay [22]. Moreover, Horsley et al. found that when comparing patients postlaparoscopic Roux‐en‐Y gastric bypass surgery, a multimodal pain regimen consisting of celecoxib and acetaminophen significantly reduced maximum pain scores and length of hospital stay compared to patients that exclusively received opioids [23].

Within the field of otolaryngology, although opioid prescription research has increased, there is limited standardization regarding postoperative multimodal pain regimens for similar procedure types. Our study is one of few to investigate the role of selective COX‐2 inhibitors in adult patients after procedures such as T&A and UPPP with tonsillectomy. In contrast with our study findings, one study found no significant reduction in pain or opioid consumption with the addition of celecoxib after tonsillectomy or T&A [24]. However, the authors proposed that 200 mg of celecoxib twice daily, which coincides with the recommended 400 mg daily dosing for acute pain, may not be a strong enough analgesic when the dose is split as described [24]. Recart et al. also previously studied this nuance of dosing and found that 400 mg celecoxib was significantly more effective than 200 mg and placebo, and that 200 and 400 mg were both significantly effective in reducing postoperative fentanyl requirements [12]. Our study design similarly divided the celecoxib dosing into two separate 200 mg doses, therefore, further investigation is warranted to test this potential difference in pain relief. Another study investigating post‐tonsillectomy pain showed no significant difference in pain scores with the addition of celecoxib; however, it demonstrated a statistically significant reduction in narcotic use and a clinically significant improvement in return of normal activity and diet [25]. Our study did not evaluate return to function, but this metric would be clinically relevant to investigate in future studies. Moreover, aside from the adult population, few studies have also evaluated the role of celecoxib in children after T&A. Notably, Giordarno et al. performed a randomized control trial that concluded celecoxib was efficacious at reducing opioid consumption, with greater than a 36% cumulative reduction in opioid use and a positive association between worsening pain and impact of celecoxib on opioid consumption [26]. Two other studies were conducted in a pediatric population, demonstrating a decrease in opioid use with the addition of celecoxib in adenotonsillectomy and a reduction in post‐T&A pain and co‐analgesic consumption, respectively [27, 28]. The conclusions of these previous studies further support the notion that celecoxib can be effective and utilized in diverse patient populations.

Alongside the efficacy of celecoxib, it is important to consider its safety and explore the potential adverse effects of adding celecoxib to patient pain regimens. Common side effects evaluated in numerous studies include general postoperative effects and those attributable to the addition of celecoxib. For example, one study recorded adverse events of postoperative nausea, vomiting, diarrhea, constipation, bleeding, and drowsiness, and found that the number of vomiting episodes was the only reported side effect significantly higher in the celecoxib group [24]. With regards to the safety profile of celecoxib specifically, numerous studies investigated bleeding risk and did not determine increased postoperative blood loss greater than the original risk post‐tonsillectomy [29, 30]. Our study did not include self‐reported adverse effects including nausea or vomiting; however, readmission and postoperative complications were evaluated and recorded. Two patients in the treatment cohort receiving celecoxib presented to the Emergency Department with postoperative bleeding necessitating cauterization in the OR, consistent with the inherent procedural bleeding risk and postoperative timeframe for bleeding to occur [31, 32]. In addition to medication adverse effects, potential drug–drug interactions within a pain regimen are also important considerations. Masclee et al. found an increased risk of upper gastrointestinal bleed with concomitant use of celecoxib and corticosteroids compared with either agent as monotherapy, although the risk was lower than that associated with corticosteroid use in combination with nonselective NSAIDs or low‐dose aspirin [33]. In our study, this was not evaluated, and no gastroprotective agents were prescribed. However, this should be considered in future directions and clinical practice when celecoxib and prednisone are used concurrently.

Limitations intrinsic to our study include the cohort study design rather than a blinded randomized control trial, the small sample size of the treatment group, and enrolled study participants from a single academic institution. These factors may affect the statistical power and generalizability of the study. This study design also raises the likelihood for several biases, including nonresponse bias, specifically of the POD 10 survey, and recall and reporting biases of patient pain scores and self‐reporting method of total opioid usage. Additionally, pain scores were not collected beyond postoperative day 10, which leaves limited insight into longer term pain control, and daily opioid consumption was not determined. Collecting data on patients' daily opioid use could provide additional insight into appropriate duration of opioid therapy and prescribing patterns. Future studies should incorporate larger sample sizes, utilize randomized controlled study designs, and include extended follow up periods to better assess pain management efficacy and monitor potential bleeding risks. Furthermore, patients were instructed to follow the standard practice of opioid disposal. This includes dropping them off at drug take back locations, or in congruence with the Food and Drug Administration (FDA) “flush list,” to dispose of leftover opioids at home [34]. Therefore, future studies should also include returning leftover opioids to the surgeon at a follow‐up appointment for nonbiased opioid measuring.

## 5 Conclusion

In this study, we examined how the addition of celecoxib to a postoperative multimodal pain regimen affected patient pain scores and opioid consumption. Patients who received celecoxib as part of their postoperative regimen consumed significantly fewer opioids within 10 days following their procedure compared to those historically who did not receive celecoxib. The addition of celecoxib did not significantly affect pain scores on POD 1 through 10, nor did it impact opioid refill rates or rates of surgical readmission. Similar results were found when stratified by specific procedure type. This study supports that celecoxib may be added to multimodal pain regimens to help decrease reliance on postoperative opioid use for surgical pain control in adults undergoing benign oropharyngeal surgery, although further investigation is warranted.

## Funding

The authors have nothing to report.

## Conflicts of Interest

The authors declare no conflicts of interest.

## Data Availability

The data that support the findings of this study are available on request from the corresponding author. The data are not publicly available due to privacy or ethical restrictions.
